# Reduction in Price of Antipyrin

**Published:** 1898-09

**Authors:** 


### THERAPEUTIC NOTES.

REDUCTION IN PRICE OF ANTIPYRIN. On account of the expiration of the patents covering the process of manufacturing this drug, the price has lately been reduced from $1.40 per ounce to as low as 30 cents per ounce. Our patent laws should be amended so as to prevent in the future this species of extortion on the part of foreign chemists on the people of this country.

